# Effect of Thermal Budget on the Electrical Characterization of Atomic Layer Deposited HfSiO/TiN Gate Stack MOSCAP Structure

**DOI:** 10.1371/journal.pone.0161736

**Published:** 2016-08-29

**Authors:** Z. N. Khan, S. Ahmed, M. Ali

**Affiliations:** 1 Advanced Electronics Labs (AEL), International Islamic University, Islamabad, Pakistan; 2 Comsats Institute of Information Technology, Islamabad, Pakistan; Gazi Universitesi, TURKEY

## Abstract

Metal Oxide Semiconductor (MOS) capacitors (MOSCAP) have been instrumental in making CMOS nano-electronics realized for back-to-back technology nodes. High-*k* gate stacks including the desirable metal gate processing and its integration into CMOS technology remain an active research area projecting the solution to address the requirements of technology roadmaps. Screening, selection and deposition of high-*k* gate dielectrics, post-deposition thermal processing, choice of metal gate structure and its post-metal deposition annealing are important parameters to optimize the process and possibly address the energy efficiency of CMOS electronics at nano scales. Atomic layer deposition technique is used throughout this work because of its known deposition kinetics resulting in excellent electrical properties and conformal structure of the device. The dynamics of annealing greatly influence the electrical properties of the gate stack and consequently the reliability of the process as well as manufacturable device. Again, the choice of the annealing technique (migration of thermal flux into the layer), time-temperature cycle and sequence are key parameters influencing the device’s output characteristics. This work presents a careful selection of annealing process parameters to provide sufficient thermal budget to Si MOSCAP with atomic layer deposited HfSiO high-*k* gate dielectric and TiN gate metal. The post-process annealing temperatures in the range of 600°C -1000°C with rapid dwell time provide a better trade-off between the desirable performance of Capacitance-Voltage hysteresis and the leakage current. The defect dynamics is thought to be responsible for the evolution of electrical characteristics in this Si MOSCAP structure specifically designed to tune the trade-off at low frequency for device application.

## Introduction

Titanium nitride (TiN) is a potential application specific material, which provides a favourable combination of physical and chemical properties for semiconductor manufacturing such as low resistance, comparatively high transmittance within the visible spectrum, hardness and chemical resistance [1]. Titanium nitride also possesses good quality mechanical, tribological, electrical, biomedical, and optical properties. The reliance of the mechanical and electrical properties of titanium nitride thin films deposited with the help of variety of techniques upon silicon substrates have been studied in past to exhibit their potential for CMOS industry driven applications [2]. In some previous studies [3–4], the TiN-Si hetrojunctions were fabricated by the deposition of TiN thin films onto the refined Si substrates by using the DC reactive magnetron sputtering. These nitrides have also shown to exhibit the property to tune the work function due to their thermal stability [5]. Currently, research on various electrode materials, starting from polycrystalline metal films to composite conducting oxides, is being carried out [5]. With TiN placed effectively as ultra-thin layer or part of the compositional stack in Si CMOS processing. The thermal stability and electrical properties of one such stack in metal oxide semiconductor (MOS) capacitors after post-metal annealing in H_2_ or N_2_+H_2_ ambient in a range of temperature 30 minutes are examined [6] and analyzed. C-V profiling for characterization of these nano-scale devices has been a standard analytical technique in manufacturing industry [7]. NMOS C-V profiling techniques (the conventional Capacitance-Voltage profiling and deep depletion (DD) C-V have also been in practice to profile the subject devices [8]. Similarly, optical characterization of titanium nitride is accomplished by means of ellipsometry, and electrical characterization is performed using C-V and I-V profiling to obtain the work function and resistivity of these thin sensitive films [6].

**Fig 1:**
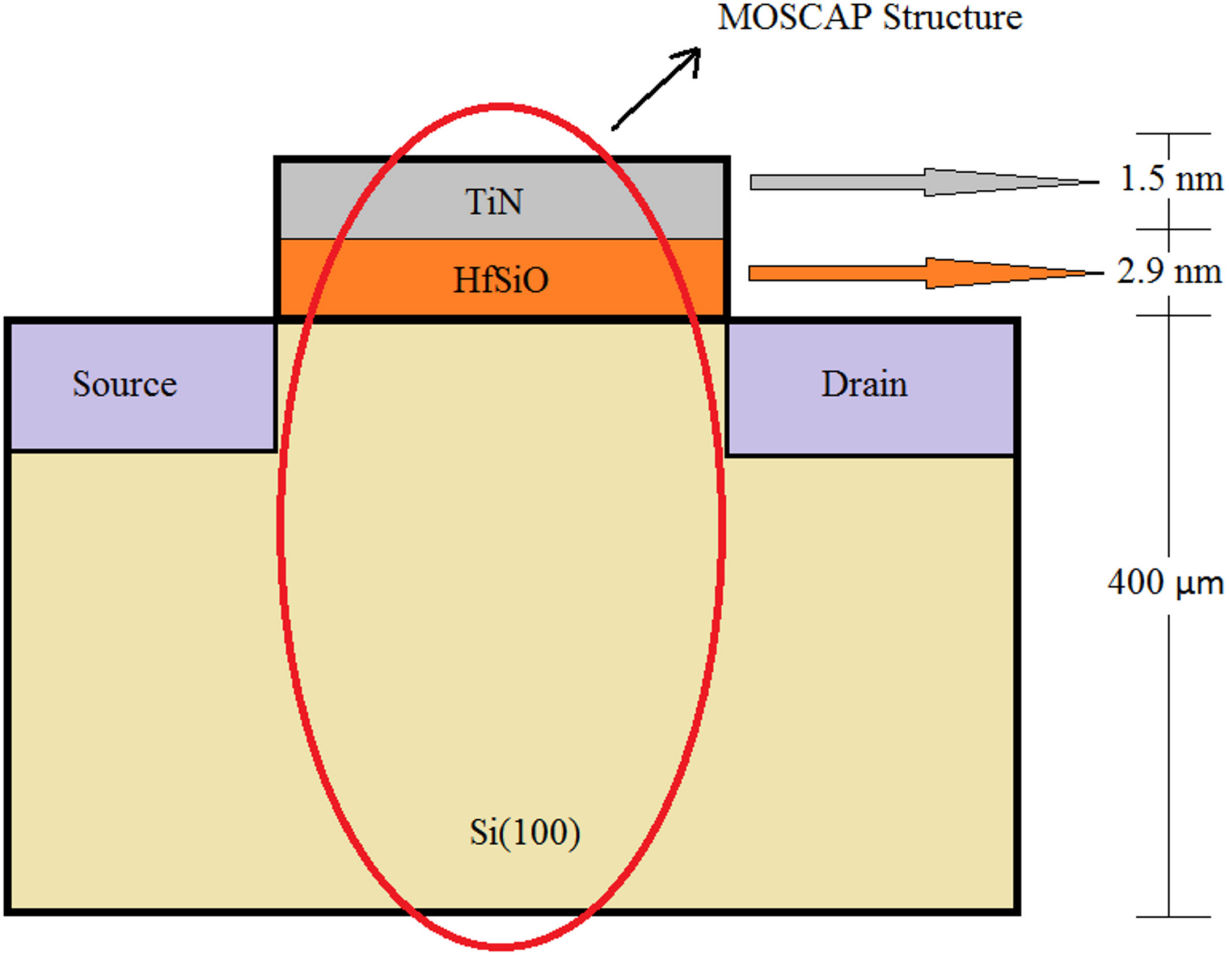
MOSCAP Structure used in this work (Device Cross Section).

In this paper, we investigate the MOSCAP structure as part of the CMOS family in terms of its sensitive electrical characteristics including detailed I-V and C-V analysis. The study is targeted towards TiN ultra-thin film as the gate electrode in the MOSCAP structure. As a substitute to the conventional gate dielectric (SiO_2_), high-*k* HfSiO is used as dielectric material beneath the gate metal for the device structure as shown in Fig 1. Hafnium Silicate has proven to be a promising dielectric material with low diffusivity of impurities and channel mobility features.

## Experimental

MOSCAP structure is fabricated by utilizing HfSiO layer as high-*k* dielectric material instead of conventional SiO_2_ knowing its competitiveness of EOT scaling. Atomic layer deposition (ALD) technique is used for the deposition of both the high-*k* dielectric HfSiO layer and TiN metal gate in NLD-4000 ALD system. The deposition chamber is preheated at 315°C and the substrate of Si (100) is placed in the reaction chamber. A high *-k* dielectric layer is deposited by injecting the Hf and Si liquid precursors one by one resulting into a proper reaction yielding a fine layer of dielectric material. Following this, TiN layer is deposited on top of the dielectric layer onto the same wafer using the same ALD technique. The chamber temperature is maintained at 400°C with TiCl_4_ and NH_3_ gases duly injected in the reaction chamber for TiN ultra-thin deposition. TiCl_4_ is injected with N_2_ which is used as a carrier gas (also acted as a purge gas) at the same time. High purity ammonia gas (~99%) is used as a reactant. The working pressure of the reaction chamber was maintained at 0.55 Torr (with ±5% of control accuracy). Detailed ALD process parameters to deposit these ultra- thin layers on Si (100) standard wafer are summarized in Table 1.

**Table 1:** Atomic Layer Deposition Process Conditions for HfSiO and TiN Ultra-Thin Layers to fabricate the device structure.

S.No	Process Material	Precursors	Non Metal precursor	Growth Rate per Cycle (at Source Temperature)	Temperature (°C)	Deposition Rate (at Source Temperature)	Pulse sequence (seconds)
1	HfSiO	TEMAH/TDMAS/O_2_ (for Hf, Si and O respectively)	O_2_	0.43 Å/cycle	300	0.24 nm/min	1/2/2/2 (Precursor/Ar Purge/O_2_ Plasma/Ar Purge)
2	TiN	TiCl_4_	NH_3_	0.3 Å/cycle	400	0.11 nm/min	2/2/2/2 (Precursor/N_2_/ NH_3_/N_2_)

All the annealing schedules are performed on the Rapid Thermal Processor (RTP) during the processing of MOSCAP structures. Rapid Thermal Processor (RTP) is known to reduce the thermal redistribution of impurities at relatively high temperature. The exposure of the samples to the heating flow for relatively smaller times also tends to reduce the probabilities of oxidation of TiN films. Post deposition annealing of HfSiO is carried out in RTP for 470°C with no ramps of temperature in between and dwelled at 30 seconds. The ambience of nitrogen is chosen to purge the environment with a cooling effect. The atomic layer deposited TiN ultra-thin layer is also subjected to RTP with temperatures intentionally chosen to be in the process window from 600°C to 1000°C for 20 seconds in each case. Again, a nitrogen gas ambience is created to anneal the samples at nominal atmospheric pressure. The electrical characteristics of the MOSCAP devices are measured using a sophisticated Automatic System for Material Electro-Physical Characterization (ASMEC) tool in the Advanced Electronics Laboratory.

## Results and Discussion

### I-V Characteristics and Leakage Resistance

**Fig 2:**
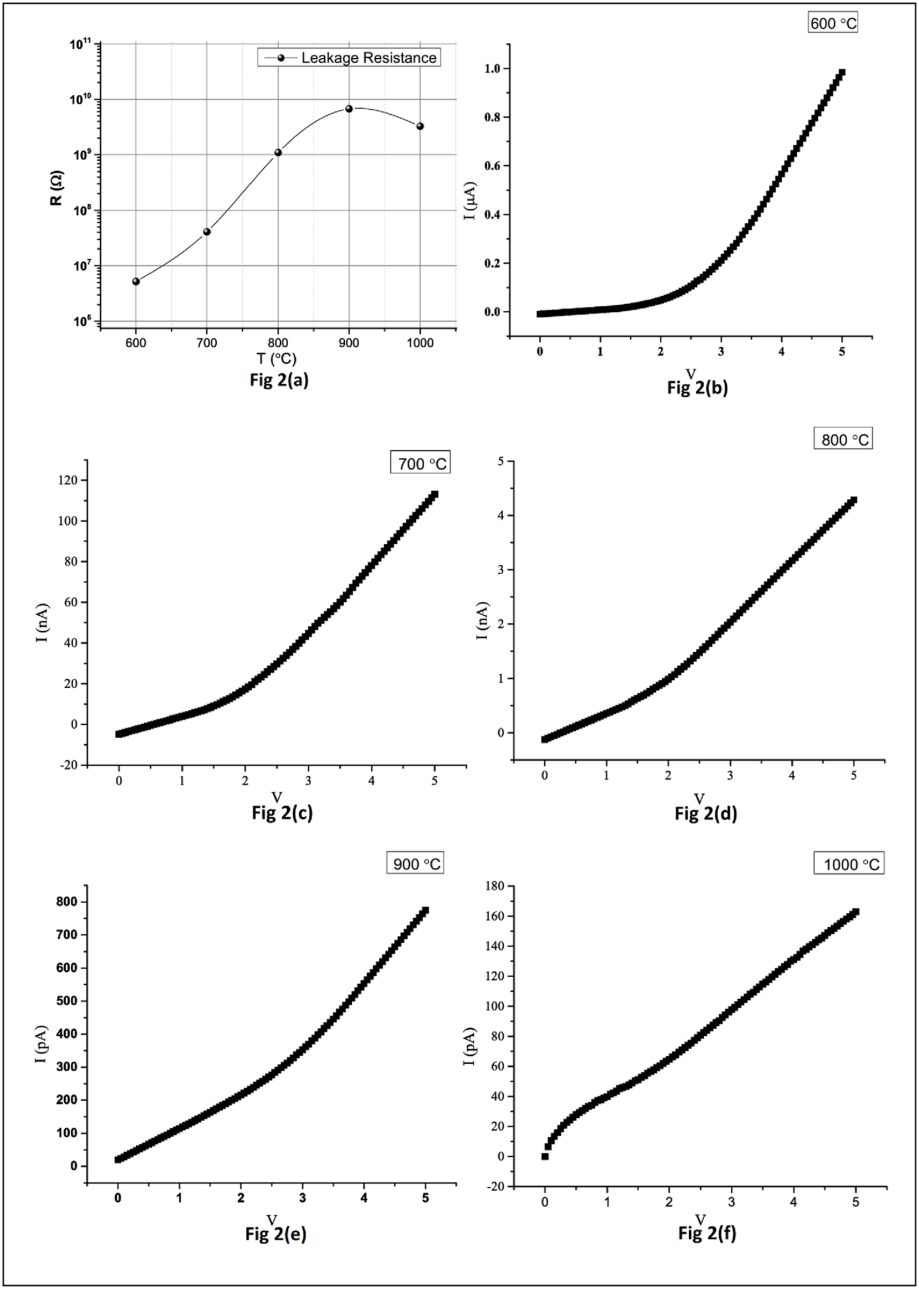
Leakage Resistance and I-V characteristics for different annealing temperatures. (a) Leakage Resistance at different annealing temperatures. (b) I-V characteristic curve for 600°C. (c) I-V characteristic curve for 700°C. (d) I-V characteristic curve for 800°C. (e) I-V characteristic curve for 900°C. (f) I-V characteristic curve for 1000°C.

Fig 2 shows the I-V profiles of the MOSCAP structure with HfSiO as dielectric material TiN and ultra-thin film as gate electrode, and subjected to relatively medium to slightly higher annealing temperatures. Fig 2, when closely examined, reveals that the leakage current is reduced with the increasing post-process annealing cycle until 900°C. A slight increase is noticed when the annealing temperature goes to 1000°C. This in turn shows an increase of leakage resistance with the increasing annealing temperatures. At relatively higher annealing temperatures (900–1000°C of process window); the change in the leakage resistance is almost negligible. The current profile, as depicted in Fig 2 is at maximum for the sample annealed at a temperature of 600°C. The obtained curves follow the pattern as in [9–10], which is a similar study for I-V curves for evaluating the metal gate electrode behavior. Our results are consistent with those obtained in [11–12] since the rise of temperature does not necessarily transform into enhanced conductivity; this phenomenon can be attributed to metal gate and dielectric interface defect states [13]. We also observe from our results that the rise of the annealing temperature also improves the linearity of the I-V correlation i.e. the linearity is much pronounced for the devices undergone the annealing temperatures of 800°C, 900°C and 1000°C in comparison to the ones with 600°C and 700°C. The possible presence of defects in the structure and randomness of the defect (vacancies in particular) interaction with the device matrix and their annihilation is thought to be responsible for the non-linearity and its gradual recovery in the signature I-V curves for various annealing conditions (Fig 2b–2f). Fig 2 also depicts that the MOSCAP structure having TiN thin layers deposited by ALD and undergone a rapid thermal process cycle at 600°C for 20 seconds does not maintain the leakage current against all bias values. For example, low voltages yield an appreciable leakage current. In other words; leakage resistance is maximum at low voltages for sample annealed at 600°C. As the voltage increases, the resistance shows a constant behavior. This provides a flexible process window to attain device output parameters at desirable bias condition.

### C-V Profiling

**Fig 3:**
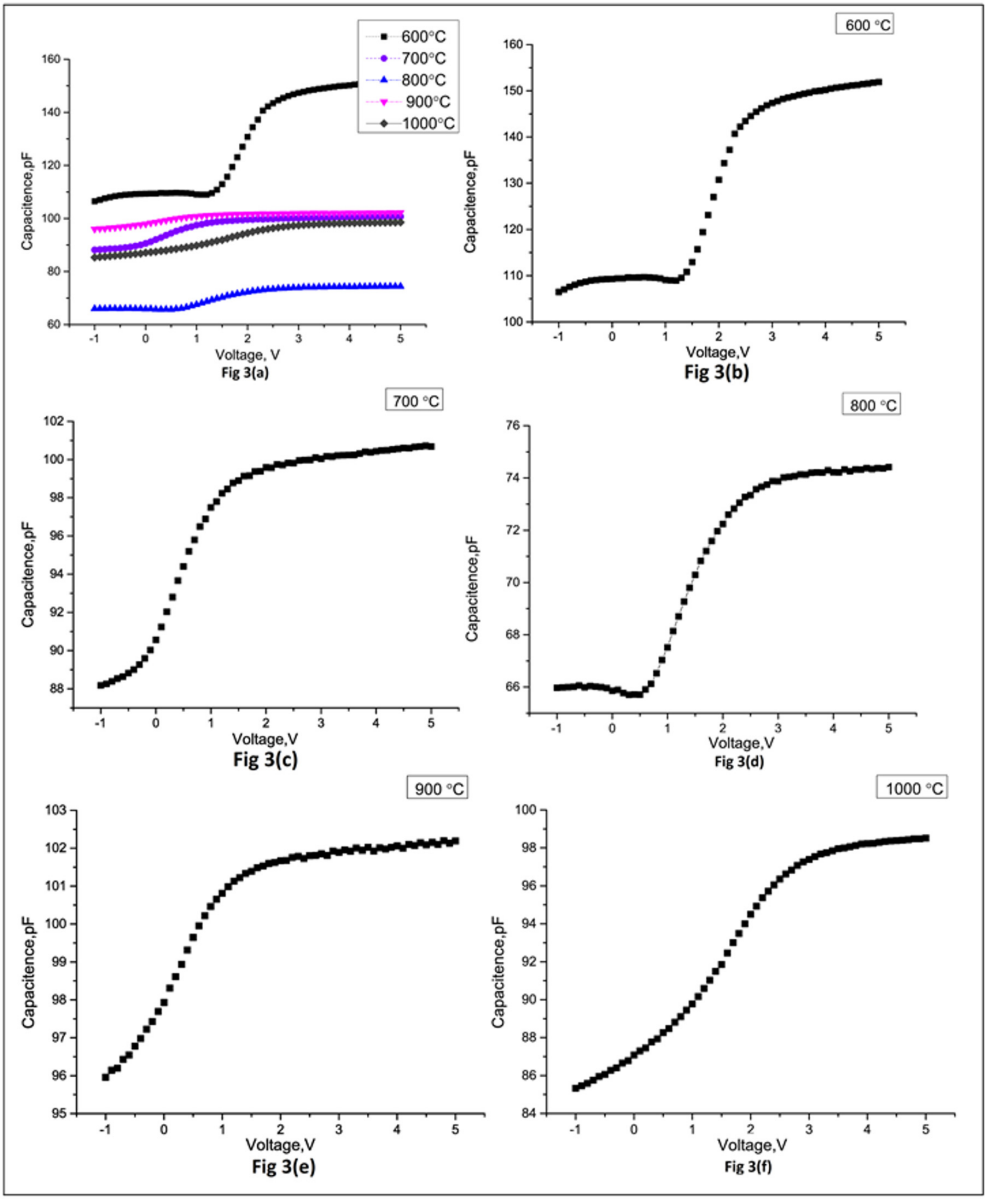
C-V Hysteresis for samples at different annealing Temperatures. (a) C-V profiles at different annealing ranging from 600 to 1000°C. (b) C-V profiling for 600°C. (c) C-V profiling for 700°C. (d) C-V profiling for 800°C. (e) C-V profiling for 900°C. (f) C-V profiling for 1000°C.

An analysis is made for the electrical characteristics of MOSCAP with TiN gate electrode at various annealing temperatures. The Capacitance-Voltage profiling is done with the help of ASMEC equipment, which is used for the electrical characterization for parameters such as I-V, C-V, QDLTS etc. The C-V profiles obtained for MOSCAP devices undergoes different annealing cycles between 600°C and 1000°C are shown in Fig 3. The C-V curves shown in Fig 3 are in close approximation with the standard curve for MOSCAP found in the literature [7]. It is evident that the values of capacitances change with the variation of the annealing cycles subjected to the MOSCAP device structure. The devices, which have undergone the rapid thermal processing of 600°C and 700°C, reflect a positive threshold voltage as compared to the ones with negative voltage for higher annealing temperatures. The threshold voltage becomes more negative as soon as the temperature rises beyond 900°C. This signifies that the slope of the representing curves (depletion in physical terms) decreases with the increase of the annealing temperatures. It is also exhibited that the samples annealed at 800°C have the lowest value of capacitance i.e. 65 pF at a voltage of about 0.5 V. The lower value of capacitance translates into lower electrostatic losses and consequently lower leakages. As the C-V profiling executed for these MOSCAP devices is performed at an operating frequency of 1.2 kHz, this may provide an estimate of the possible presence of different kinds of defects and interstitial states present in the structure. The trends are somewhat similar to an earlier study carried out at low frequencies [12]. The increase in capacitance especially at low frequency is likely to be attributed to the presence of interstitial state at Si/SiO_2_ interface in conventional MOSCAP structures [13]. The dangling bonds, humps, stretch out oxygen vacancies during deposition are also reported to play a significant role in the shift of flat band voltage for such devices [14]. The post process annealing has known influence on the dynamic re-structuring of defects and is reported to improve the mobility and threshold slopes for equivalent devices [14], such as in our case.

### Doping Profile and Built in Potential

**Fig 4:**
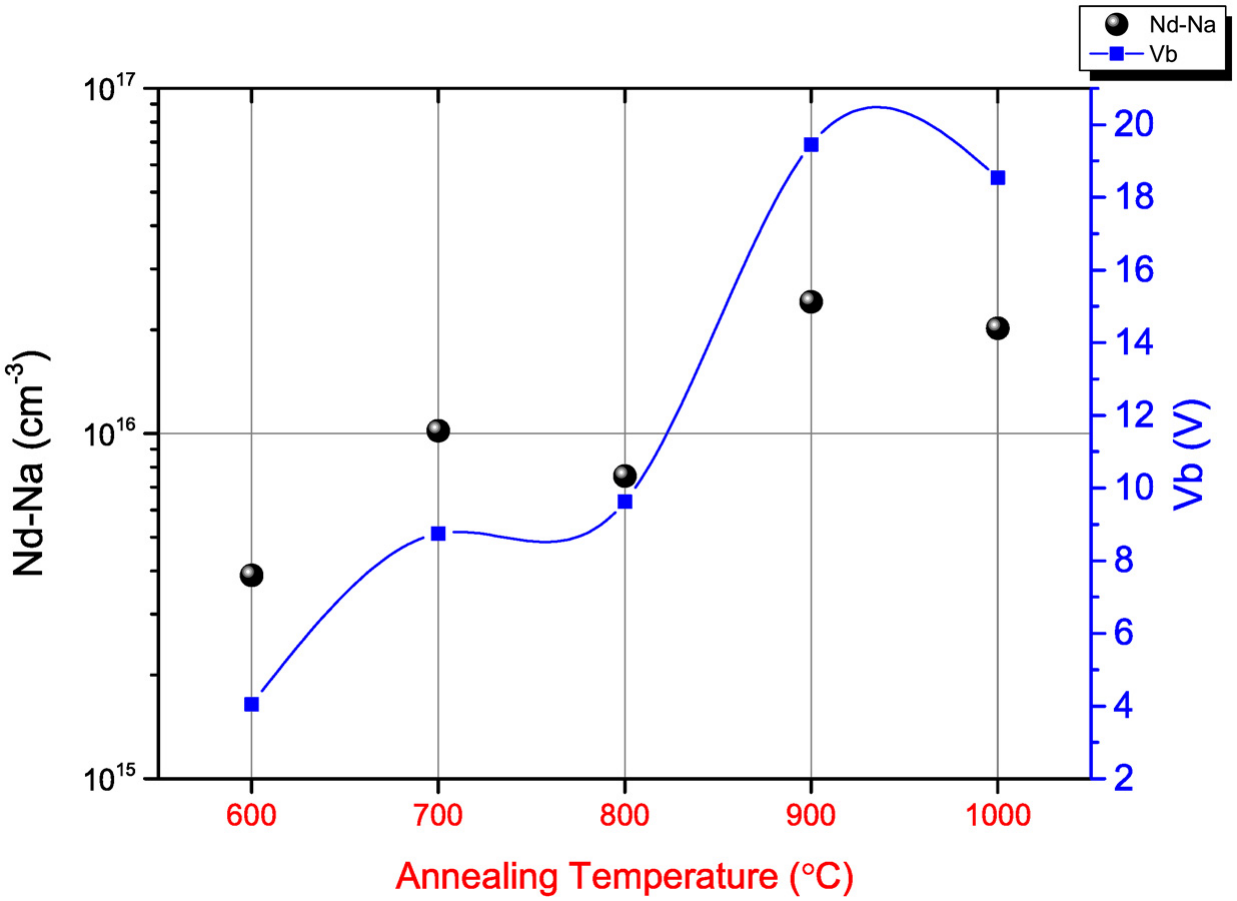
Doping profile and Built in voltage as a function of Annealing Temperature.

Fig 4 explains a correlation between the post-process annealing temperature with the effective doping profile (N_d_-N_a_) and the built-in potential for MOSCAP with ultra-thin TiN metal gate structured with ALD. The doping profile N_d_-N_a_ is visible to be highest for samples annealed at 900°C. The built in voltage V_b_ is also the highest for the 900°C annealed samples owing to the higher annealing temperature drawing out relatively larger number of majority carriers from the lattice.

The built in voltage depends directly on the number of dopants in such a way that higher number of dopant atoms yield a concentrated doping profile and a wider depletion region. Consequently, the higher built in voltage is achieved. The higher annealing temperature, as expected, results in fewer interfaces state densities and hence the probability of having defects decreases in the interface state and consequently lesser energy is required to vacate any carrier implying energy efficiency [9].

## Conclusion

We fabricated specifically designed MOSCAP structures, for possible integration with CMOS technology, with atomic layer deposited HfSiO and TiN thin layers as high-*k* dielectric and metal gate, respectively. The devices were subjected to post deposition and post-metal annealing cycles with carefully chosen rapid thermal process parameters. Low frequency electrical characterization was performed in order to tune the trade-off between the C-V hysteresis and the leakage current at the gate to facilitate design an effective energy efficient CMOS nano-electronics. It is found that the profiles obtained after different annealing temperatures are shown to closely follow the standard C-V curve for MOSCAP at low frequencies. The largest value (voltage) of the knee of the curve signifying transition from accumulation to depletion is for a sample annealed at 600°C. The slope (depletion) of the electrical profile of the device structure is found to decrease with the increasing rapid thermal annealing temperature. The leakage resistance reflects a maximum value at low voltages with almost a constant behavior with increasing bias. The threshold voltages are proving to be more negative at relatively higher annealing temperatures. The optimized device output characteristic window provides a trade-off between the desirable electrical entities such as C-V hysteresis, leakage current, flat-band voltage and leakage resistance, with the control of process conditions during the annealing kinetics. This may prove to be a way forward to design energy efficient CMOS electronics at desirable bias.

## Supporting Information

S1 Fig MOSCAP Structure used in this work (Device Cross Section). (TIF)

S2 Fig Leakage Resistance and I-V characteristics for different annealing temperatures. (TIF)

S3 Fig Leakage Resistance and I-V characteristics for 600°C–1000°C. (TIF)

S4 Fig I-V characteristic curve for 600°C. (TIF)

S5 Fig I-V characteristic curve for 700°C. (TIF)

S6 Fig I-V characteristic curve for 800°C. (TIF)

S7 Fig I-V characteristic curve for 900°C. (TIF)

S8 Fig I-V characteristic curve for 1000°C. (TIF)

S9 Fig C-V Hysteresis for samples at different annealing Temperatures. (TIF)

S10 Fig C-V profiles for samples at annealing temperatures (600°C–1000°C). (TIF)

S11 Fig C-V profiling for samples at 600°C. (TIF)

S12 Fig C-V profiling for samples at 700°C. (TIF)

S13 Fig C-V profiling for samples at 800°C. (TIF)

S14 Fig C-V profiling for samples at 900°C. (TIF)

S15 Fig C-V profiling for samples at 1000°C. (TIF)

S16 Fig Doping profile and Built in voltage as a function of Annealing Temperature. (TIF)

S1 Table Atomic Layer Deposition Process Conditions for HfSiO and TiN Ultra-Thin Layers to fabricate the device structure. (DOCX)
