# Supplementary figures and images for: Effect of Thermal Budget on the Electrical Characterization of Atomic Layer Deposited HfSiO/TiN Gate Stack MOSCAP Structure
Source: PLoS One. 2016 Aug 29;11(8):e0161736. doi: 10.1371/journal.pone.0161736 (PMC5003375; doi:10.1371/journal.pone.0161736)

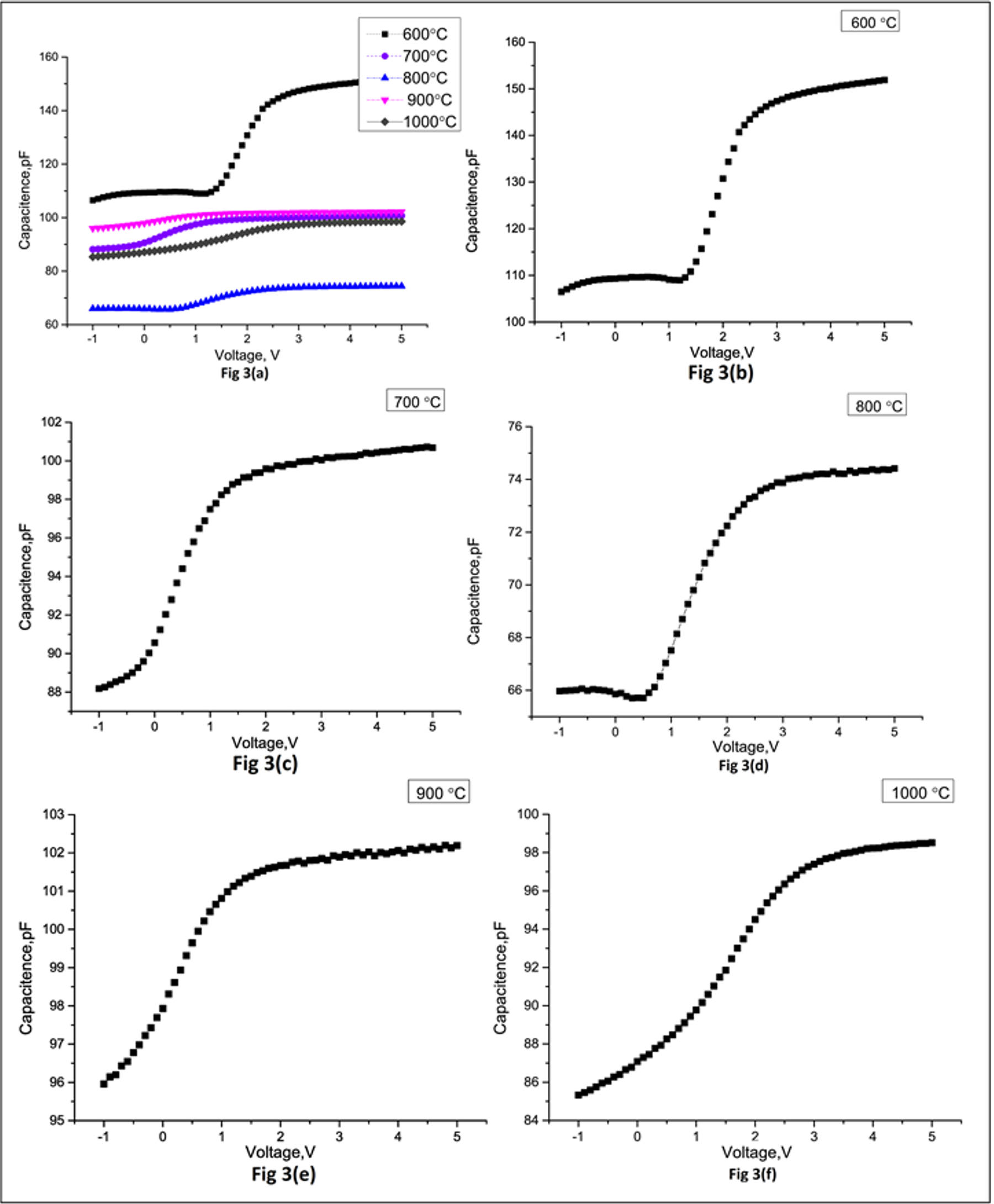

Supplement: S9 Fig — (TIF) [file pone.0161736.s009.tif]

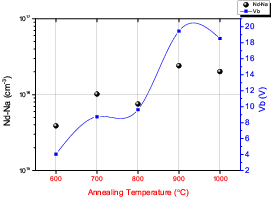

Supplement: S16 Fig — (TIF) [file pone.0161736.s016.tif]
