# Supplementary material for: Effect of Thermal Budget on the Electrical Characterization of Atomic Layer Deposited HfSiO/TiN Gate Stack MOSCAP Structure
Source: PLoS One. 2016 Aug 29;11(8):e0161736. doi: 10.1371/journal.pone.0161736 (PMC5003375; doi:10.1371/journal.pone.0161736)
Supplement: S1 Table — (DOCX) [file pone.0161736.s017.docx]

| **S.No** | **Process Material** | **Precursors** | **Non Metal precursor** | **Growth Rate per Cycle (at Source Temperature)** | **Temperature (°C)** | **Deposition Rate (at Source Temperature)** | **Pulse sequence (seconds)** |
| --- | --- | --- | --- | --- | --- | --- | --- |
| 1 | HfSiO | TEMAH/TDMAS/O_2_ (for Hf, Si and O respectively) | O_2_ | 0.43 Å/cycle | 300 | 0.24 nm/min | 1/2/2/2 (Precursor/Ar Purge/O_2_ Plasma/Ar Purge) |
| 2 | TiN | TiCl_4_ | NH_3_ | 0.3 Å/cycle | 400 | 0.11 nm/min | 2/2/2/2 (Precursor/N_2_/ NH_3_/N_2_) |
